# Contrast induced nephropathy among patients with normal renal function undergoing coronary angiography

**DOI:** 10.15171/jrip.2016.05

**Published:** 2016-02-26

**Authors:** Ahmadreza Assareh, Saeed Yazdankhah, Shahla Majidi, Nasim Nasehi, Seyed Seifollah Beladi Mousavi

**Affiliations:** ^1^Department of Cardiology, Faculty of Medicine, Ahvaz Jundishapur University of Medical Sciences, Ahvaz, Iran; ^2^Chronic Renal Failure Research Center, Faculty of Medicine, Ahvaz Jundishapur University of Medical Sciences, Ahvaz, Iran

**Keywords:** Contrast nephropathy, Coronary angiography, Diabetes mellitus

## Abstract

**Introduction:** Although contrast induced nephropathy (CIN) is a well-known complication of radiocontrast media administration among patients with underlying renal insufficiency, however the data about CIN among patients with normal renal function are few and it seems that CIN often remained under-diagnosed among these patients.

**Objectives:** The aim of present study was evaluation of CIN in diabetic and nondiabetic patients with normal renal function undergoing coronary angiography.

**Patients and Methods:** This cross-sectional and prospective study has conducted on patients with normal renal function candidate for diagnostic coronary angiography at Imam hospital, Ahvaz, Iran from October 2010 to February 2011. CIN defined as an increase in serum creatinine (sCr) >0.5 mg/dL after two days of contrast administration. A standardized questionnaire was used to collect demographics, clinical and laboratory data.

**Results:** A total of 254 patients (140 males and 114 Females with mean age of 56.6 ± 11.9 years) were included in the study. Of them, 60 patients (23.6%) had congestive heart failure (CHF) and 57 patients (22.4%) had diabetes mellitus (DM). The mean sCr levels before contrast administration in men and women were 1.05 ± 0.22 and 0.93 ± 0.17 mg/dL respectively. In overall CIN occurred in 27 patients (10.6%) with no difference between males and females (*P* = 0.386) and in patients with or without CHF (*P* = 0.766). There was a significant association between CIN and DM (*P* = 0.001) and mean volume of contrast administration (*P * = 0.001).

**Conclusion:** Although CIN is a common problem in patients with diabetic nephropathy undergoing coronary angiography, diabetic patients without diabetic nephropathy and also patients without DM who had normal renal function are also at risk of contrast nephropathy.

**Implication for health policy/practice/research/medical education:**

Contrast induced nephropathy (CIN) is a well-known complication of radiocontrast media administration among patients with underlying renal insufficiency, however the data about CIN among patients with normal renal function are few and it seems that CIN often remained under-diagnosed among these patients.

## Introduction

The use of radiocontrast media has increased greatly from the past decades for diagnostic radiography and interventional procedures and it is estimated that approximately 60 million people in the world are used radiocontrast media each year (1).

On the other hand, the administration of radiocontrast media may lead to acute renal failure (ARF) that begins soon after the contrast is administered. Contrast induced nephropathy (CIN) is defined as an impairment of renal function characterized by an increase in plasma creatinine of more than 25% or 0.5 mg/dL above baseline in the absence of an alternative etiology (2-7). The incidence of CIN is variable and rangesfrom 0% to over 50% in various studies. This variability results from differences in the presence or absence of patient-related and contrast-related risk factors, the definition of CIN, prospective or retrospective determination of incidence, and the exact radiologic procedure (4-9). In the vast majority of patients, the renal failure induced by contrast agents, is nonoliguric, mild and transient and it typically begins within the first 12 to 24 hours after the contrast administration and the recovery of renal function occurs within three to five days (3-7).

However, the severe and persistent renal failure with a peak rise in the plasma creatinine that exceeds 5 mg/dL may also happen especially among patients with preexisting underlying kidney disease (5-11).

Although CIN is a well-known complication of radiocontrast media administration among patients with underlying renal insufficiency, however the data about CIN among patients with normal renal function are few and it seems that CIN often under-diagnosed among these patients.

## Objectives

The aim of present study was evaluation of CIN in diabetic and nondiabetic patients with normal renal function undergoing coronary angiography.

## Patients and Methods

### Study patients

This cross-sectional and prospective study has conducted on patients with normal renal function candidate for diagnostic coronary angiography at Imam hospital, Ahvaz, Iran from October 2010 to February 2011.

A standardized questionnaire was used to collect demographic data, the record of previous disease and drugs, dose and kinds of prescribed radiocontrast media, vital signs, and laboratory data results including serum creatinine (sCr) and blood urea nitrogen (BUN) before and after of the contrast administration. A day before and then one and two consecutive days after coronary angiography, blood samples were obtained and analyzed for BUN and sCr, using the commercial kits. CIN was defined as an increase in the sCr concentration equal or more than 0.5 mg/dL after one or two days of contrast administration.

Patients with the following characteristics were excluded from the study; female or male patients, who had sCr above 1.4 and 1.2 mg/dL respectively. Those who had allergic reaction to the contrast agents, patients who had received angiotensin inhibitor, diuretic and or nephrotoxic agents like aminoglycoside drugs, those with a history of significant systemic diseases like respiratory disease and or hepatic failure. Patients who were need to cardiopulmonary resuscitation during angiography, those who had hemodynamic instability during angiography, patients with cardiogenic shock and pulmonary edema and finally patients who received angiotensin inhibitors, diuretics and/or nephrotoxic agents like aminoglycosides.

### Ethical issues

1) The research followed the tenets of the Declaration of Helsinki; 2) the nature of the study was explained to the participants and written informed consents were obtained from them. They were free to leave the study at any time and 3) research was approved by the ethical committee of chronic renal failure research center of Jundishapur University of Medical Sciences, Ahvaz, Iran.

### Statistical analysis

We used SPSS version 15 software for statistical analysis. Continuous variables with normal distribution were expressed as mean ± SD and statistical significance was assessed at* P*<0.05.

## Results

**Figure 1:**
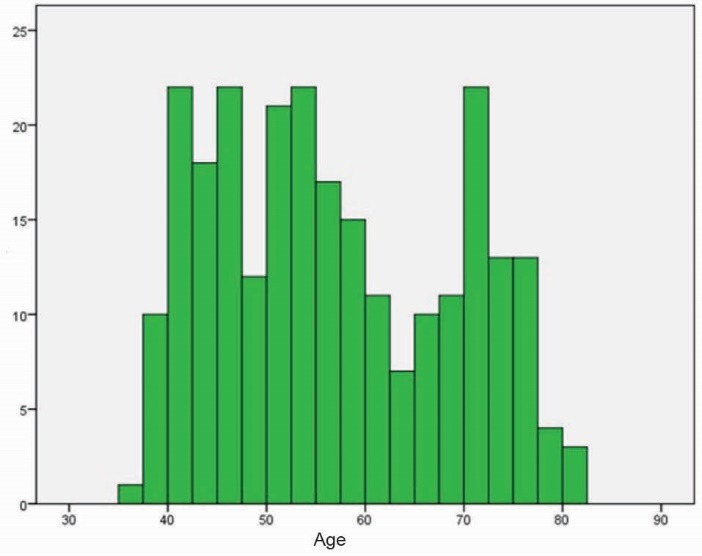
Age distribution of patients.

In overall, the population of our study was 280 patients. Of them, 24 patients excluded from the study, and finally 254 patients (140 males and 114 females with mean age of 56.6±11.9 years) were enrolled to the study. The youngest and the oldest patients had 37 and 82 years old respectively. Figure 1 shows the age distribution of patients.

Sixty patients (23.6%) had congestive heart failure (CHF), 57 patients (22.4%) had diabetes mellitus (DM) and 12 patients (4.7%) had DM and CHF concomitantly. The mean sCr levels before contrast administration in men and women were 1.05±0.22 and 0.93 ±0.17 respectively (*P*=0.75). Overall CIN occurred in 27 (10.6%) patients (17 males and 10 females) with no difference between males and females (*P*=0.386) and in patients with and without CHF (*P*=0.766). The mean volume of contrast administered was directly and significantly associatedwith the incidence of nephrotoxicity. The volume of contrast was higher among patients developingCIN (90±31.2 ml) than patients who did not (71±25.2 ml, *P*=0.001).

In multivariateanalysis, each 10cc rises of injected contrast material, was associated with an incremental odds ratio of10.20 (*P*<0.001).In our study, a significant association between CIN and DM was seen (*P*=0.001).

## Discussion

It is well established that the incidence of CIN is higher among patients with chronic kidney disease (CKD) (12-19). For example Rihal et al evaluated the incidence of acute kidney injury (AKI) among 7586 patients undergoing percutaneous coronary intervention. The results of the study showed that the overall incidence of contrast nephropathy which was defined as an increase in the sCr concentration more than 0.5 mg/dL is 3.3%. However the incidence of AKI among patients with CKD was higher and the magnitude of the risk was directly associated with the decline in glomerular filtration rate. It was 22% and 31% among patients with a sCr of 2.0 to 2.9 mg/dL and equal or more than 3 mg/dL respectively (12).

The results of other studies including Parfrey et al (13), Schwab et al (14), Manske et al (15), Lautin et al (16), Barrett et al (17), Rudnick et al (18), Mehran et al (19) are also demonstrated that the patients with CKD especially diabetic patients are at higher risk for contrast nephropathy.

In contrast to patients with underlying renal insufficiency, it seems that contrast nephropathy often under-diagnosed among patients with normal renal function.

The results of our study showed that the prevalence of contrast nephropathy among patients who have a baseline sCr less than 1.4 and 1.2 mg/dL in men and women and are undergoing coronary angiography is high and contrast nephropathy occurred in 10.6% of our patients.

The incidence of contrast nephropathy is significantly higher in our study compared to the results of Parfrey et al and Rudnick et al studies (13,18). As an example, in a randomized trial, Rudnick et al evaluated the incidence of contrast nephropathy among 341 patients with sCr ≤1.5 mg/dl, who received iohexol during percutaneous interventions. The results of this study showed that the risk of contrast nephropathy among patients with normal renal function is less than 1% (18).

In contrast to the results of our study which showed, a significant association between contrast nephropathy and DM among patients who have normal renal function, Rudnick et al reported no significant difference in the incidence of contrast nephropathy between diabetics and nondiabetic patients without preexisting underlying kidney disease.

High total dose of contrast agent was another risk factor of contrast nephropathy in our study and the mean volume of contrast was significantly associatedwith the incidence of contrast nephropathy. Other studies including Cigarroa et al (20), Lautin et al (16), Barrett et al (17), Rudnick et al (18) and McCullough et al (21) have also demonstrated a dose-dependent risk of renal dysfunction similar to the results of our study. According to the results of these studies, the risk of contras nephropathy is increased as the volume of contrast agents are increased and lower doses of contrast are safer, but not free of risk (16-21).

It is important to note that low dose of contrast has been variably defined, generally ranging from less than 30 ml of contrast agent in some studies to less than 125 ml of contrast material in the other studies (11,12,16,17). It is also important to note that diabetic patients with advanced diabetic nephropathy may be at risk of contrast nephropathy from as little as 20 to 30 mcc of contrast agent (15).

## Conclusion

CIN is a well-known complication of radiocontrast media administration among patients with underlying renal insufficiency, however the data about CIN among patients with normal renal function are few and it seems that CIN often under-diagnosed among these patients. The results of our study showed that the prevalence of contrast nephropathy among patients who have normal renal function is also high and contrast nephropathy occurred in significant percent of patients.

## Limitations of the study

The limitation of our study was small sample size and therefore, a multicenter clinical trial with large sample size is needed for better evaluation.

## Acknowledgments

The authors wish to thank the dean of cardiology trans­plantation center of Ahvaz Jundishapur University of Medical Sciences for his help in data collection.

## Authors’ contribution

All authors participated equally in conducting the research and preparing of the manuscript.

## Conflicts of interest

The authors declared no competing interests.

## Ethical considerations

Ethical issues (including plagiarism, data fabrication, double publication) have been completely observed by the authors.

## Funding/Support

This paper is issued from thesis of Nasim Nasehi (Thesis number # U-89224) and financial support was provided by the Chronic Renal Failure Research Center of Ahvaz Jundishapur University of Medical Sciences.

## References

[1] Morcos SK, Thomsen HS, Webb JA (1999). Contrast-media-induced nephrotoxicity: a consensus report. Eur Radiol.

[2] Weisbord SD, Palevsky PM (2005). Radiocontrast-induced acute renal failure. J Intensive Care Med.

[3] Aspelin P, Aubry P, Fransson SG, Strasser R, Willenbrock R, Berg KJ (2003). Nephrotoxic effects in high-risk patients undergoing angiography. N Engl J Med.

[4] Asif A, Epstein M (2004). Prevention of radiocontrast-induced nephropathy. Am J Kidney Dis.

[5] Barrett BJ, Parfrey PS (1994). Prevention of nephrotoxicity induced by radiocontrast agents. N Engl J Med.

[6] Conlon PJ, Little MA, Pieper K, Mark DB (2001). Severity of renal vascular disease predicts mortality in patients undergoing coronary angiography. Kidney Int.

[7] Rudnick MR, Berns JS, Cohen RM, Goldfarb S (1994). Nephrotoxic risks of renal angiography: contrast media-associated nephrotoxicity and atheroembolism--a critical review. Am J Kidney Dis.

[8] Beladi-Mousavi SS, Bashardoust B, Nasri H, Ahmadi A, Tolou-Ghamari Z, Hajian S (2014). The theme of the world diabetes day 2014; healthy living and diabetes; a nephrology view point. J Nephropharmacol.

[9] Beladi Mousavi SS, Nasri H, Rafieian-Kopaei M, Tamadon MR (2012). Metformin improves diabetic kidney disease. J Nephropharmacol.

[10] Tamadon MR, Beladi-Mousavi SS (2013). Erythropoietin; a review on current knowledge and new concepts. J Renal Inj Prev.

[11] Beladi Mousavi SS, Alemzadeh Ansari MJ, Cheraghian B (2011). Outcome of Patients on Hemodialysis in Khuzestan, Iran. NDT Plus.

[12] Rihal CS, Textor SC, Grill DE (2002). Incidence and prognostic importance of acute renal failure after percutaneous coronary intervention. Circulation.

[13] Parfrey PS, Griffiths SM, Barrett BJ, Paul MD, Genge M, Withers J (1989). Contrast material-induced renal failure in patients with diabetes mellitus, renal insufficiency, or both A prospective controlled study. N Engl J Med.

[14] Schwab SJ, Hlatky MA, Pieper KS, Davidson CJ, Morris KG, Skelton TN (1989). Contrast nephrotoxicity: a randomized controlled trial of a nonionic and an ionic radiographic contrast agent. N Engl J Med.

[15] Manske CL, Sprafka JM, Strony JT, Wang Y (1990). Contrast nephropathy in azotemic diabetic patients undergoing coronary angiography. Am J Med.

[16] Lautin EM, Freeman NJ, Schoenfeld AH, Bakal CW, Haramati N, Friedman AC (1991). Radiocontrast-associated renal dysfunction: incidence and risk factors. AJR Am J Roentgenol.

[17] Barrett BJ, Parfrey PS, Vavasour HM, McDonald J, Kent G, Hefferton D (1992). Contrast nephropathy in patients with impaired renal function: high versus low osmolar media. Kidney Int.

[18] Rudnick MR, Goldfarb S, Wexler L, Ludbrook PA, Murphy MJ, Halpern EF (1995). Nephrotoxicity of ionic and nonionic contrast media in 1196 patients: a randomized trial The Iohexol Cooperative Study. Kidney Int.

[19] Mehran R, Aymong ED, Nikolsky E, Lasic Z, Iakovou I, Fahy M (2004). A simple risk score for prediction of contrast-induced nephropathy after percutaneous coronary intervention: development and initial validation. J Am Coll Cardiol.

[20] Cigarroa RG, Lange RA, Williams RH, Hillis LD (1989). Dosing of contrast material to prevent contrast nephropathy in patients with renal disease. Am J Med.

[21] McCullough PA, Wolyn R, Rocher LL, Levin RN, O’Neill WW (1997). Acute renal failure after coronary intervention: incidence, risk factors, and relationship to mortality. Am J Med.

